# Mechanical characteristics analysis and control algorithm for floating raft system with mass variation

**DOI:** 10.1038/s41598-023-36661-9

**Published:** 2023-06-10

**Authors:** Liang Shi, Guanghui Cheng, Wenjun Bu

**Affiliations:** 1 Institute of Noise and Vibration, Naval University of Engineering, Wuhan, China; 2 National Key Laboratory On Ship Vibration and Noise, Wuhan, China

**Keywords:** Engineering, Electrical and electronic engineering, Mechanical engineering

## Abstract

The ship floating raft system adopts the integrated design of large liquid tanks and rafts, which can optimize the arrangement in the cabin and increase the intermediate mass of the system to achieve efficient vibration isolation of equipment. One of the major challenges is that the change of liquid mass in the tank will cause displacement of the raft, which will change the modal characteristics of the system and affect the stability of the vibration isolation system performance. This paper establishes a mechanical analysis model of a floating raft system under time-varying liquid mass conditions. Taking a ship variable mass floating raft system as the research object, the effect of mass change on the characteristics of raft displacement, isolator load distribution, and modal frequency of the vibration isolation system is analyzed. The analysis shows that when the liquid tank goes from full load to no-load state, its mass change accounts for 40% of the total mass of the raft, which will cause a large displacement of the raft and change the low order modal frequency of the system, bringing the risk of equipment safety and vibration isolation performance degradation. Therefore, an adaptive variable load control method is proposed to realize the raft attitude balance and load equalization optimization under the variable mass condition of the floating raft air spring system. The test results show that the proposed control method can automatically adapt to the large mass gradual change from full load to no load of the liquid tank on the raft, and control the displacement of the raft structure from about 10 mm to 1.5 mm, which effectively ensures the stability of the air spring system performance.

## Introduction

In contemporary times, advanced ships have widely adopted the large whole cabin floating raft technology to ensure efficient vibration isolation of integrated power equipment^1–4^. To further optimize the overall arrangement and enhance the floating raft’s vibration isolation and impact resistance, the concept of a floating raft vibration isolation system with a liquid tank design has been proposed^5^. This involves rigidly fixing a large mass liquid tank to the raft, which significantly increases the mass ratio and rigidity of the system, resulting in efficient vibration isolation and improved impact safety. Liu^6^ delved into the dynamic characteristics of the integrated liquid tank and raft structure, examining the effects of different loading rates of the liquid tank on the system’s natural frequency and isolation effect. Although changes in the rigid body mode and natural frequency do not significantly differ under various loading rates due to the small proportion of the weight of the liquid tank, the weight of the tank remains beneficial in enhancing the vibration isolation effect near the low-frequency resonance frequency. Furthermore, L. Zhiyang^7^ established a dynamic model for a floating raft vibration isolation system with a liquid tank, analyzing the influence of the mass effect of the liquid medium, tank form, structural stiffness, and loading rate of the tank volume on the floating raft system's acoustic performance. The findings indicate that when the floating raft structure has sufficient stiffness, its acoustic performance is significantly improved as the tank loading rate increases in the relevant low-frequency range.

**Figure 1:**
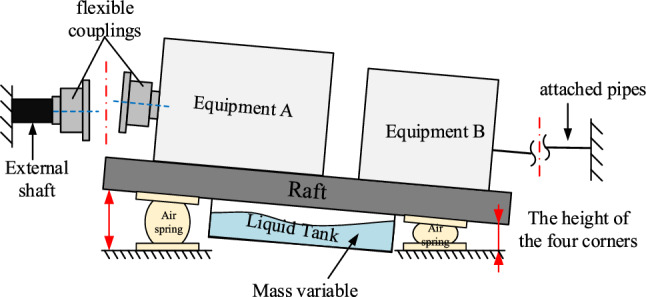
The schematic diagram of the floating raft air spring system for time-varying mass.

However, in the above-integrated structure, the gradual change of mass brought about by liquid consumption will cause changes in the load, working height, and the system stiffness characteristics of the raft, resulting in problems such as isolator bias load, height overshoot, deterioration of equipment alignment and excessive pipeline deformation^8–11^ as illustrated in Fig. 1. In addressing the issue of uniform load carrying and attitude balance of vibration isolator systems for large power equipment of ships, Zuo^12^ expanded the solutions from the perspective of raft design. He^13^ proposed an intelligent air spring technology to reduce the dependence of vibration isolator design on parameters, such as equipment weight, the center of gravity, and load characteristics by using the variable load adjustment capability of air springs. For the problem of external load disturbance during the use of vibration isolation devices, a dynamic model of the floating raft air spring system is established, the motion characteristics under the external disturbance force are analyzed, and a raft attitude balance control algorithm is proposed to ensure the efficient vibration isolation performance of the system^14,15^. The above research results are all based on the assumption of small disturbance, using constant system analysis and control methods to solve the problem of design optimization and external load disturbance control of a large air spring system, which does not apply to large mass time-varying floating raft vibration isolation systems.

Regarding mass time-varying system problems, various scholars have conducted related studies from diverse fields. Ma^16^ focuses on the dynamic characteristics of containers with fuel consumption. With fuel consumption, the fuel container system undergoes vibrations leading to decreased mass. This decrease in mass results in increased system vibration frequencies and induces additional negative damping. This additional negative damping is proportional to the rate of mass change and slows the vibration decay. Wu^17^ researched the influence of propellant consumption on the dynamic properties of a large-scale satellite structure. The analysis outcomes indicate that the frequency of the main mode, as well as the frequency and amplitude of response, increases with propellant consumption, while the frequency of the local mode remains unchanged. These studies focused on the problem of variable mass factors on the system influence, with varying effects guiding system control methods. Cao^18^ considers the influence of variable mass effect and accurately predicts the dynamic response of maglev-turning electric spindles for ensuring cutting stability while realizing the expected effect of magnetic bearing control accuracy. Similarly, Li^19^ proposes a novel dynamic model for underwater vehicles with variable mass and center of gravity to solve the problem of disturbances caused by mass and center of gravity variation. The variable mass system is simulated in the case of mining, which reveals that variations of the center of gravity, weight, and moment inertia are not appropriate to regard as disturbances. Deng^20^ developed a mathematical model for spacecraft with large liquid propellant tanks wobbling and depleting. The study combines sliding mode control with an adaptive algorithm to control position and attitude maneuvers, and numerical simulation results confirm that the proposed controller is effective for a spacecraft with liquid propellant stored in two parallel tanks.

This paper delves into investigating the floating raft air spring system under a wide range of variable mass conditions. The study establishes a mechanical analysis model under variable mass conditions and proposes a control method to adapt to time-varying mass perturbations. The control objective is to maintain key performance parameters, such as displacement, load distribution, and natural frequency of the variable mass floating raft structure, in a stable state to achieve reliable vibration isolation effects.

The rest of this paper is organized into four parts. Section “Mechanical characteristics analysis” analyzes the mechanical characteristics of the variable-mass floating raft vibration isolation system, establishes a system dynamics model, and studies the influence of variable-mass factors on the modal characteristics and attitude characteristics of the floating raft. In Section “Control method”, the paper outlines the design of the control method and adaptive algorithm, which are categorized into calculation module, pressure control module, displacement control module, and uniform load optimizer. The experimental results and analyses are presented in Section “Experiment and analysis”. The section provides an in-depth understanding of the system's response under variable mass conditions and validates the effectiveness of the proposed control method. Finally, some concluding remarks are presented.

## Mechanical characteristics analysis

### System description

In this paper, a type of ship floating raft with the liquid tank air spring system is used as the research object to carry out the system load, raft attitude, and vibration modal analysis under variable mass conditions. The parameters are shown in Table 1, and the air spring used is rated at 8 tons, and its placement form is shown in Fig. 3.

**Table 1:** Parameters of the analysis model of the raft air spring system.

Parameters	Notation	value
Vertical static stiffness of air spring Transverse static stiffness of air spring	$\eta_{r}$	2.0 kN/mm/MPa
	$\eta_{p} ,\eta_{q}$	4.8 kN/mm/MPa
Natural frequency of air spring	$f_{s}$	4.3 Hz
Rated load working pressure of air spring	$p$	2.0 MPa
Structure weight under full load	$M_{1}$	160 T
Structure weight under no load	$M_{0}$	80 T
Center of gravity coordinates under full load	$s_{{M_{1} }}^{x} ,s_{{M_{1} }}^{y} ,s_{{M_{1} }}^{z}$	(0, 0, 0)mm
Center of gravity coordinates under no load	$s_{{M_{0} }}^{x} ,s_{{M_{0} }}^{y} ,s_{{M_{0} }}^{z}$	(0, 73, 161)mm

**Figure 2:**
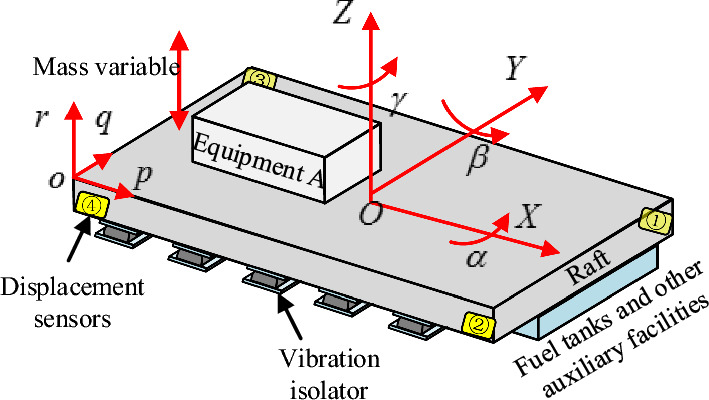
Sketch of the coordinate system of the floating raft air spring system.

For a certain type of ship floating raft with a liquid tank system, with the center of gravity of the raft and its equipment at full load as the coordinate origin, the overall coordinate system of the vibration isolation system is established, as shown in Fig. 2. The raft size (L × W × H) is 5.3 × 3.2 × 1.5 m; 20 air springs are placed symmetrically along the port and starboard sides of the raft to support the weight of the raft with the equipment. At the same time, four displacement sensors are arranged between the raft and the base at the four corners to monitor the height change of the raft and the base.

### Dynamical model

The integrated structure of the raft and liquid tank has greater rigidity. For quasi-static problems (such as load distribution of air spring system, attitude response of raft ) and low-order modal analysis, think of the raft as a rigid body, the equations of motion of the variable mass system can be established as Refs.^21,22^:

1 $$ \left( {{\mathbf{M}}_{r} + {\mathbf{M}}_{t} \left( t \right)} \right){\mathbf{\ddot{X}}}_{g} { + }\left( {{\mathbf{C}} + {\dot{\mathbf{M}}}_{t} \left( t \right)} \right){\dot{\mathbf{X}}}_{g} + {\mathbf{K}}({\mathbf{p}}){\mathbf{X}}_{g} = {\mathbf{F}}_{t} . $$

In the formula, ${\mathbf{M}}_{r}$ is the mass matrix of the invariant structure, ${\mathbf{M}}_{t} \left( t \right)$ is the mass matrix of the variable mass structure, ${\mathbf{C}}$ is the Rayleigh damping matrix, ${\mathbf{K}}({\mathbf{p}})$ is the system stiffness matrix and related to the air spring operating pressure ${\mathbf{p}}$, ${\mathbf{X}}_{{\text{g}}} = [x_{g} ,y_{g} ,z_{g} ,\alpha ,\beta ,\gamma ]^{{\text{T}}}$ is the translational and rotational movement of the raft center of gravity in the X, Y, and Z coordinate directions, and ${\mathbf{F}}_{t}$ is the external disturbance of the system including inertial forces generated by the mass change.

The system stiffness matrix can be expressed as:

2 $$ {\mathbf{K}}\left( {\mathbf{p}} \right) = \sum\limits_{i = 1}^{N} {({\mathbf{G}}_{i}^{{}} )^{T} {\mathbf{T}}_{i}^{T} {\mathbf{k}}\left( {p_{i} } \right){\mathbf{T}}_{i}^{{}} {\mathbf{G}}_{i}^{{}} } , $$

where, ${\mathbf{G}}_{i}^{{}}$ is the position matrix of the i-th air spring in the overall coordinate system, ${\mathbf{T}}i$ is the rotation matrix, and its elements are the cosine values of the angle between each main elastic axis of the isolator and the overall coordinate axis of the system. ${\mathbf{k}}\left( {p_{i} } \right)$ is the airbag vibration isolator stiffness, which can be expressed under small deformation conditions as:

3 $$ {\mathbf{k}}\left( {p_{i} } \right) = p_{i} {\text{diag}} \left( {\eta_{r} ,\eta_{p} ,\eta_{q} } \right), $$

n the formula (3), $\eta_{r} ,\eta_{p} ,\eta_{q}$ represents the linear coefficient of pressure versus stiffness in $r,p,q$ direction for the air spring.

4 $$ {\mathbf{G}}_{i}^{{}} = \left[ {\begin{array}{*{20}c} 1 & 0 & 0 & 0 & {s_{i}^{z} } & { - s_{i}^{y} } \\ 0 & 1 & 0 & { - s_{i}^{z} } & 0 & {s_{i}^{x} } \\ 0 & 0 & 1 & {s_{i}^{y} } & { - s_{i}^{x} } & 0 \\ \end{array} } \right], $$

5 $$ {\mathbf{T}}i = \left[ {\begin{array}{*{20}c} {\lambda_{px}^{i} } & {\lambda_{py}^{i} } & {\lambda_{pz}^{i} } \\ {\lambda_{qx}^{i} } & {\lambda_{qy}^{i} } & {\lambda_{qz}^{i} } \\ {\lambda_{rx}^{i} } & {\lambda_{ry}^{i} } & {\lambda_{rz}^{i} } \\ \end{array} } \right], $$

where $s_{i}^{x}$, $s_{i}^{y}$ and $s_{i}^{z}$ are the three coordinate components of the center point of the top of air spring i-th in the overall coordinate system. According to the relationship between the vibration isolator coordinate system and the overall coordinates in Fig. 2, the small angular variation generated by the displacement of the raft structure is neglected as:

6 $$ {\mathbf{T}}i = \left[ {\begin{array}{*{20}c} 0 & 1 & 0 \\ 1 & 0 & 0 \\ 0 & 0 & 1 \\ \end{array} } \right]. $$

Since the liquid mass is consumed slowly and can be seen as a quasi-static problem, the acceleration and velocity terms in Eq. (1) can be ignored when analyzing the effect of mass change on the attitude of the raft, and the displacement at the center of gravity is obtained as follows:

7 $$ {\mathbf{X}}_{g} \left( t \right) = {\mathbf{K}}\left( {\mathbf{p}} \right)^{ - 1} {\mathbf{F}}_{t} $$

Then for any point of the raft the motion displacement ${\mathbf{X}}_{i}$ with respect to the hull foundation can be described by ${\mathbf{X}}_{{\text{g}}}$ and the position matrix ${\mathbf{G}}_{i}^{{}}$, which combined with Eq. (7) can be expressed as:

8 $$ {\mathbf{X}}_{i} = {\mathbf{G}}_{i} {\mathbf{K}}\left( {\mathbf{p}} \right)^{ - 1} {\mathbf{F}}_{t} . $$

### Analysis of modal characteristics influence

**Figure 3:**
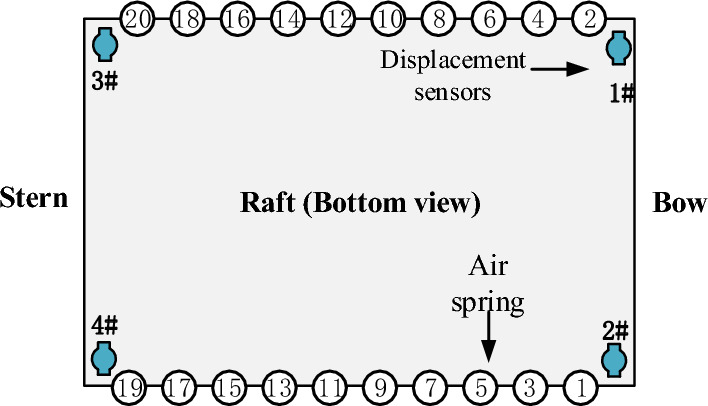
Arrangement diagram of air spring.

According to the weight and center of gravity parameters of the floating raft structure under full load and no-load conditions in Table 1, and the schematic diagram of air spring arrangement in Fig. 3, the optimal pressure distribution of the air spring is ${\mathbf{p}}_{f}$ and ${\mathbf{p}}_{e}$ respectively according to the principle of equal load, as shown in Table 2, the vibration isolator and system stiffness can be obtained by the formula (2) and (3).

**Table 2:** Load distribution of air spring under full load and no-load conditions.

Air spring ID	Q1/Q2	Q3/Q4	Q5/Q6	Q7/Q8	Q9/Q10	Q11/Q12	Q13/Q14	Q15/Q16	Q17/Q18	Q19/Q20
Full-load pressure ${\mathbf{p}}_{f}$ (MPa)	1.94	1.94	1.94	1.95	1.95	1.95	1.95	1.95	1.96	1.96
No-load pressure ${\mathbf{p}}_{e}$ (MPa)	1.21	1.20	1.19	1.18	1.18	1.17	1.16	1.14	1.14	1.13

The liquid is considered as an additional mass of the integrated structure and analyzed in ANSYS using the wet modal method to simulate the variable mass characteristics of the liquid. The upper equipment is considered a concentrated mass and the low-order modal analysis of the vibration isolation system is performed. Table 3 gives the first 12 orders of modal vibration of the system, the three columns of modal frequencies corresponding to the system mass and pressure distribution are Case 1: full load-${\mathbf{p}}_{f}$, Case 2: no load-${\mathbf{p}}_{f}$ and Case 3: no load-${\mathbf{p}}_{e}$. Case 2 refers to the process from full load to no load without adjusting the air spring pressure, and the air spring stiffness remains unchanged; Case 3 means that the system has carried out variable load control according to the mass change, and the air spring pressure is adjusted to ${\mathbf{p}}_{e}$ at no load case.

**Table 3:** Effects of Mass Changes on System Modes.

Modal category	Modal order	Modal characteristics	Case 1	Case 2	Case 3
The rigid body modes	1	Longitudinal rocking vibration	2.79	3.72	2.64
	2	Vibrate vertically up and down	3.52	4.96	3.52
	3	Lateral rocking vibration	3.61	5.70	4.05
	4	Longitudinal rocking vibration	5.78	7.63	5.44
	5	Lateral rocking vibration	6.35	7.70	5.46
	6	Vertical torsional vibration t	7.37	9.08	6.47
The elastic modes	7	1st order vertical bending	27.79	28.24	28.19
	8	Torsional vibration	37.10	46.32	45.71
	9	Vertical 1st-order bending and torsion coupling	40.60	49.59	49.22
	10	2nd order vertical bending	77.55	121.92	121.57
	11	1st order lateral bending	83.41	122.59	122.25
	12	Transverse 1st-order bending-torsional vibration coupling	90.40	128.08	127.69

Contrast analysis of the modal frequencies from Case 1 to Case 2 shows that from full load to no load, as the liquid mass decreases, the first 6 orders of rigid body modal frequencies increase slightly, about 1 ~ 2 Hz, while the 8 ~ 12 orders of elastic modal frequencies move significantly to high frequencies. The data of case 3 shows that the variable load control of air spring pressure can reduce the low-order mode frequency variation, and the first 6-order mode frequency variation range is within 1 Hz and biased towards low-frequency movement, while the elastic mode frequency is not significantly affected by the adjustment of air spring pressure.

**Figure 4:**
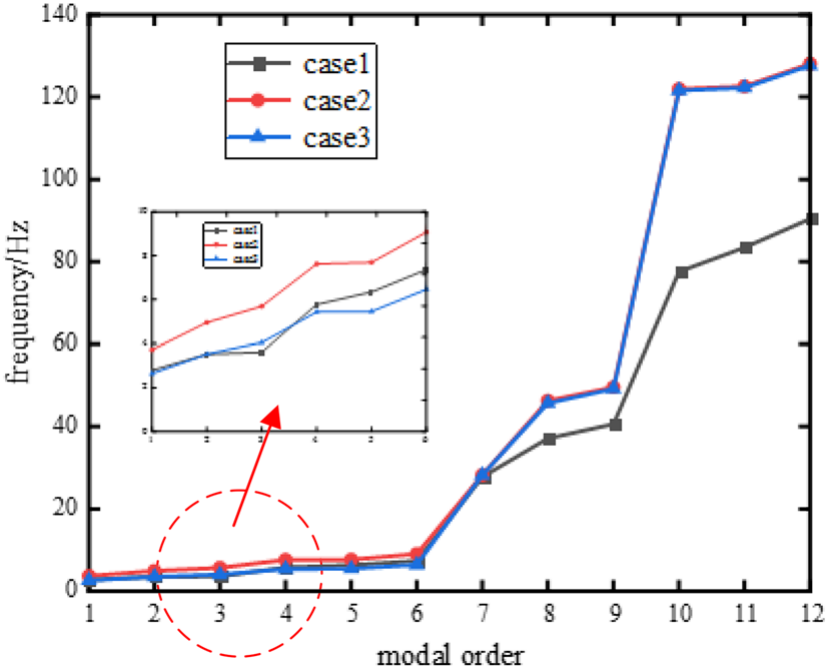
Natural frequency varying with the consuming of mass.

Figure 4 displays more visually the first 12 orders of modal frequencies of the floating raft under the three cases. It can be seen that in the variable-mass floating raft design, the lower-order mode of the system can be adjusted by the adapt air spring working pressure to keep the same; while the elastic mode is greater influenced by the change of liquid mass. Therefore, the excitation frequency of the main vibration source equipment should be considered and avoid the entire range of modal frequency variations under variable mass.

### Analysis of floating raft attitude effects

The attitude of the floating raft is mainly affected due to the disturbance force generated from the change of mass, then the disturbance force can be expressed as:

9 $$ {\mathbf{F}}_{m}^{{}} = {\mathbf{G}}_{m}^{T} \left( {0,0,mg} \right)^{T} - {\mathbf{G}}_{M1}^{T} \left( {0,0,M_{1} g} \right)^{T} , $$

where ${\mathbf{G}}_{m}^{T}$ and ${\mathbf{G}}_{M1}^{T}$ are the center of gravity matrix of the raft under current loading mass $m$ and full loading mass $M1$, respectively. Since the symmetrical design of the liquid tank ensures that the liquid mass on the raft is symmetrically distributed along the Y-axis, $s_{m}^{x} = 0$ in position matrix ${\mathbf{G}}_{m}^{{}}$ in any loading state, Eq. (9) can be simplified as:

10 $$ \begin{gathered} {\mathbf{F}}_{m}^{{}} = \left( {\begin{array}{*{20}c} 0 & 0 & {mg - M_{1} g} & {s_{m}^{y} mg} & 0 & 0 \\ \end{array} } \right)^{T} \hfill \\ s_{m}^{y} = \frac{{M_{1} - m}}{{M_{1} - M_{0} }}s_{{M_{0} }}^{y} . \hfill \\ \end{gathered} $$

**Figure 5:**
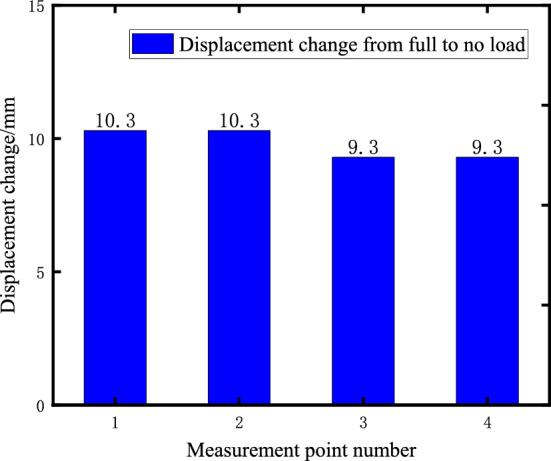
Displacement change of measuring point with full load to no-load conditions.

Calculated according to Table 1 parameters from full load to no load conditions, the change of the raft four corners 1 ~ 4 vertical displacement measurement points as shown in Fig. 5.

It can be seen that from full load to no load, with the consumption of the mass on the raft, the maximum vertical deformation of the air spring reaches 10.3 mm, which exceeds the displacement compensation capacity of the external elastic joint and affects work safety. Therefore, it is necessary to adjust the load and stiffness of the air spring system to reduce the displacement of the raft structure.

## Control method

### Control response model

By adjusting the air spring pressure, a part of the disturbance force caused by the mass change can be offset and the raft vertical displacement response can be reduced. The force generated by the airbag pressure adjustment can be expressed as:

11 $$ {\mathbf{F}}_{i}^{{}} = {\mathbf{G}}_{i}^{T} \left( {0,0,\Delta p_{i} A_{e} } \right)^{T} , $$

where $A_{e}$ is the effective bearing area of air spring rated height, and $\Delta p_{i}$ is the i-th air spring pressure adjustment amount. According to Eq. (8) and (11), it is possible to calculate the displacement response at any measured point j caused by the pressure adjustment of the i-th air spring:

12 $$ \Delta {\mathbf{X}}_{ij} = \Delta p_{i} A_{e} {\mathbf{G}}_{j} {\mathbf{K}}\left( {\mathbf{p}} \right)^{ - 1} {\mathbf{G}}_{i}^{T} \left( {0,0,1} \right)^{T} . $$

According to Eqs. (2) and (3), the system stiffness matrix after pressure adjustment can be expressed as Eq. (13), which is convenient for iterative calculation of the control system.

13 $$ \begin{gathered} {\mathbf{K}} = {\mathbf{K}}\left( {\mathbf{p}} \right) + p_{i} {\mathbf{A}}_{i} \hfill \\ {\mathbf{A}}_{i} = p_{i} ({\mathbf{G}}_{i}^{{}} )^{T} {\mathbf{T}}_{i}^{T} {\text{diag}} \left(\eta_{r} ,\eta_{p} ,\eta_{q} \right){\mathbf{T}}_{i}^{{}} {\mathbf{G}}_{i}^{{}} \hfill \\ \end{gathered} $$

According to Eq. (12), the displacement response of points 1# ~ 4# under the action of any air spring unit pressure change can be calculated. Since the air spring pressure adjustment mainly affects the vertical displacement of the raft, the vertical displacement component $\Delta {\mathbf{X}}_{ij}$ of $\Delta z_{ij}$ is taken. Then the vertical displacement response caused by the ith air spring when the pressure adjustment is increased by 1 bar can be expressed as ${\mathbf{r}}_{i} { = }\left( {\Delta z_{i1} ,\Delta z_{i2} ,\Delta z_{i3} ,\Delta z_{i4} } \right)^{T}$, Pressure control response matrix of the whole vibration isolation system is given as:

14 $$ {\mathbf{R}}{ = }\left( {{\mathbf{r}}_{1} ,{\mathbf{r}}_{2} , \cdots ,{\mathbf{r}}_{N} } \right), $$

where N is the number of air springs. Since the vibration isolator and displace sensors are symmetrically arranged along the Y-axis, the control response ${\mathbf{R}}_{1} = ({\mathbf{r}}_{1} ,{\mathbf{r}}_{3} , \cdots ,{\mathbf{r}}_{N - 1} )$ of the port and the control response ${\mathbf{R}}_{2} = ({\mathbf{r}}_{2} ,{\mathbf{r}}_{4} , \cdots ,{\mathbf{r}}_{N} )$ of the starboard have the following approximate relationship under the condition of no significant bias load.

15 $$ {\mathbf{R}}_{2} { = }\left[ {\begin{array}{*{20}c} 0 & 1 & 0 & 0 \\ 1 & 0 & 0 & 0 \\ 0 & 0 & 0 & 1 \\ 0 & 0 & 1 & 0 \\ \end{array} } \right]{\mathbf{R}}_{1} $$

**Figure 6:**
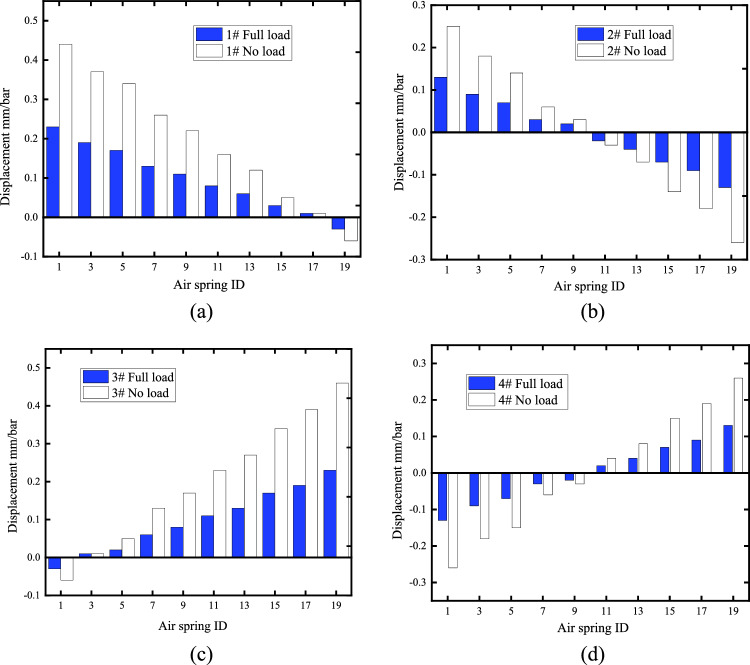
Raft displacement response with air spring inflation (**a**) Displacement response of point 1#; (**b**) Displacement response of point 2#; (**c**) Displacement response of point 3#; (**d**) Displacement response of point 4#.

With the pressure distribution ${\mathbf{p}}_{e}$ and ${\mathbf{p}}_{f}$ under full load and no-load conditions in Table 2, the control responses of the air spring (single number) inflated on the port side were calculated, respectively, as shown in Fig. 6.

It can be observed that the displacement response characteristics of the raft are mainly determined by the air spring position matrix ${\mathbf{G}}_{i}^{{}}$, the measurement point position matrix ${\mathbf{G}}_{j}$ and the system stiffness matrix ${\mathbf{K}}$. The closer the air spring is to the location of the measurement point, the greater the displacement response of the measurement point; in the no-load condition, the system stiffness is smaller and the displacement response amplitude is larger compared to the full load.

### Control method

To reduce the displacement of the raft structure and the deformation of the air spring brought by the change of mass, the vibration isolation system needs to adjust the pressure of the air spring adaptively, and at the same time, for the purpose of keeping the stability of the system performance, each air spring should try to Maintain even load or load uniform change.

**Figure 7:**
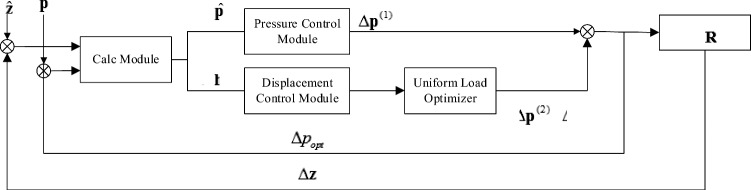
control flow chart.

The main control process consists of four parts: calculation module, pressure control module, displacement control module, and uniform load optimizer, as shown in Fig. 7.

#### Calculation module

The calculation module completes the calculation of air spring deformation ${\mathbf{h}}$, target pressure ${\hat{\mathbf{p}}}$, system stiffness ${\mathbf{K}}$ and control response ${\mathbf{R}}$.${\mathbf{K}}$ and ${\mathbf{R}}$ are calculated in Eqs. (12), (13) and (14). According to the principle of plane coordinate transformation, the air spring deformation ${\mathbf{h}}$ can be obtained from the four corners vertical displacements ${\mathbf{z}}$ of the raft as fellow:

16 $$ {\mathbf{h}} = {\mathbf{T}}_{s} {\mathbf{z}}, $$

where ${\mathbf{T}}_{s}$ is the N × 4 coordinate transformation matrix. Obviously, in the equilibrium state of the raft ${\mathbf{z}} = {\mathbf{0}}$ and ${\mathbf{h}} = {\mathbf{0}}$. To obtain the target pressure ${\hat{\mathbf{p}}}$, the actual current support force for each air spring needs to be calculated as:

17 $$ F_{i} = p_{i} A_{e} - h_{i} p_{i} \eta_{r} , $$

where $p_{i} A_{e}$ is the support force of the air spring without deformation and $- h_{i} p_{i} \eta_{r}$ is the support force provided by the air spring deformation. Assuming that the load $F_{i}$ on the ith air spring remains unchanged and returns to a deformation-free operating state, $h_{i} = 0$ , the expression with respect to the target pressure 3 is:

18 $$ \begin{gathered} F_{i} = \hat{p}_{i} A_{e} \hfill \\ \hat{p}_{i} = p_{i} \,\left( {1 - \frac{{h_{i} \eta_{r} }}{{A_{e} }}} \right). \hfill \\ \end{gathered} $$

##### Pressure control module

When $\left\| {\mathbf{z}} \right\|_{\infty } > \varepsilon_{p}$ ($\varepsilon_{p} > 0$ is the trigger pressure control threshold), this indicates that the floating raft vibration isolation system cannot provide a load that matches the current mass of the liquid tank and that the raft attitude has deviated significantly from the equilibrium position. At present, the pressure control module is invoked to adjust the air spring load.

19 $$ \Delta {\mathbf{p}}^{(1)} = {\hat{\mathbf{p}}} - {\mathbf{p}}, $$

where $\Delta {\mathbf{p}}^{(1)} = \left( {\Delta p_{1} ,\Delta p_{2} , \cdots ,\Delta p_{N} } \right)^{T}$ is the adjustment amount of each air spring pressure. To avoid control overshoot, only one airspring is selected for adjustment at a time and priority is given to the one with the largest adjustment.

#### Displacement control module

When $\left\| {\mathbf{z}} \right\|_{\infty } \ge \varepsilon_{z}$ ($\varepsilon_{z} > 0$ is the control convergence threshold) and $\left\| {\mathbf{z}} \right\|_{\infty } \le \varepsilon_{p}$. It shows that the floating raft vibration isolation system basically matches the current mass of the liquid tank, but fails to achieve attitude balance accuracy. The displacement control module should be invoked to adjust the displacement of the four corners of the raft to within the accuracy range. At this point, based on the stiffness ${\mathbf{K}}$ and control response ${\mathbf{R}}$ of the floating raft isolation system obtained by the calculation module, and then with the adjustment unit of 1 bar, the effect of each air spring pressure control on the attitude of the raft is found as follows.

20 $$ \Delta {\hat{\mathbf{z}}}_{i} = \Delta {\mathbf{z}} \pm {\mathbf{r}}_{i} , $$

where the "$\pm$" indicates the direction of pressure adjustment, " + ${\mathbf{r}}_{i}$" is the displacement response for a pressure increase of 1 bar, and "-${\mathbf{r}}_{i}$" is the displacement response for a pressure decrease of 1 bar.

According to the raft attitude convergence condition in Eq. (21), filter out all solution sets $\Delta {\mathbf{p}}^{(2)}$. $\Delta {\mathbf{p}}^{(2)}$ is an N-dimensional vector with all elements of 1, -1, or 0, indicating that the corresponding N air springs pressure should be raised by 1 bar, lowered by 1 bar, or not adjusted.

21 $$ \left\| {\Delta {\mathbf{z}}_{i} } \right\|_{\infty } - \left\| {\Delta {\hat{\mathbf{z}}}_{i} } \right\|_{\infty } > \delta \, \left( {i = 1,2, \cdots ,N} \right){,} $$

where $\delta$ affects the convergence speed and control accuracy of the raft attitude. The higher the $\delta$, the faster the convergence speed, but the control accuracy decreases. Therefore, the actual control requirements and control response characteristics of the floating raft vibration isolation system should be taken into account to determine the value of $\delta$.

#### Uniform load optimizer

**Figure 8:**
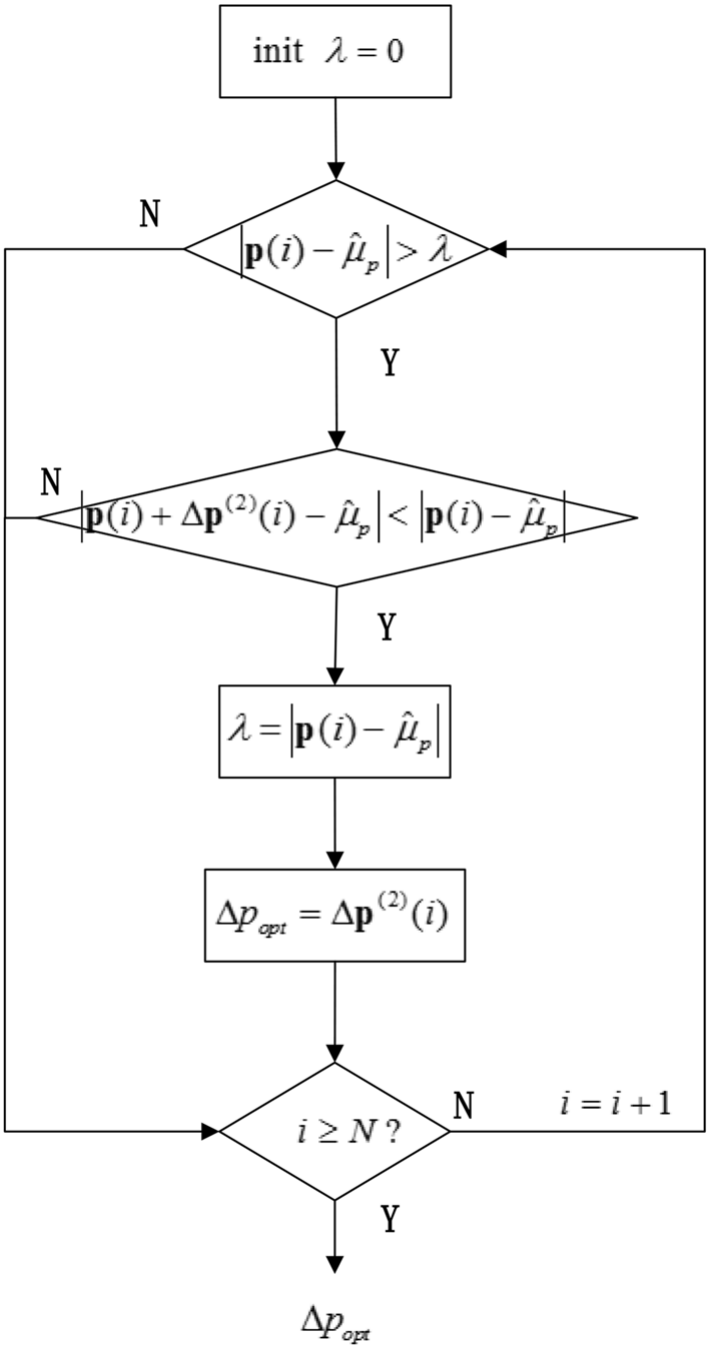
Flow Chart of Uniform Load Optimizer.

The uniform load optimizer takes the average value $\hat{\mu }_{p}$ of ${\hat{\mathbf{p}}}$ as the target and screens the elements in $\Delta {\mathbf{p}}^{(2)}$ that can reduce the maximum $\left| {{\mathbf{p}}(i) - \hat{\mu }_{p} } \right|$ to achieve the uniform load optimization of the system, which is shown in Fig. 8.

## Experiment and analysis

**Figure 9:**
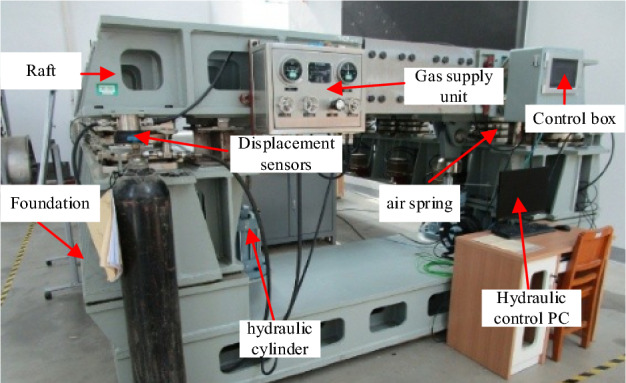
Floating raft air spring system test platform.

The test platform in this paper is mainly composed: of a raft structure, an air spring vibration isolation system, and a set of hydraulic loading systems. The raft adopts a splice structure design, the size is 5.3 × 3.2 × 1.5 m, the weight is about 10 tons, and the raft structure is supported by 20 air springs with a rated load capacity of 8 tons. A set of electro-hydraulic servo systems and synchronous loading of four actuating cylinders were adopted on the test platform to simulate the variable mass condition of the raft and realize different attitude changes of the raft, as shown in Fig. 9.

**Figure 10:**
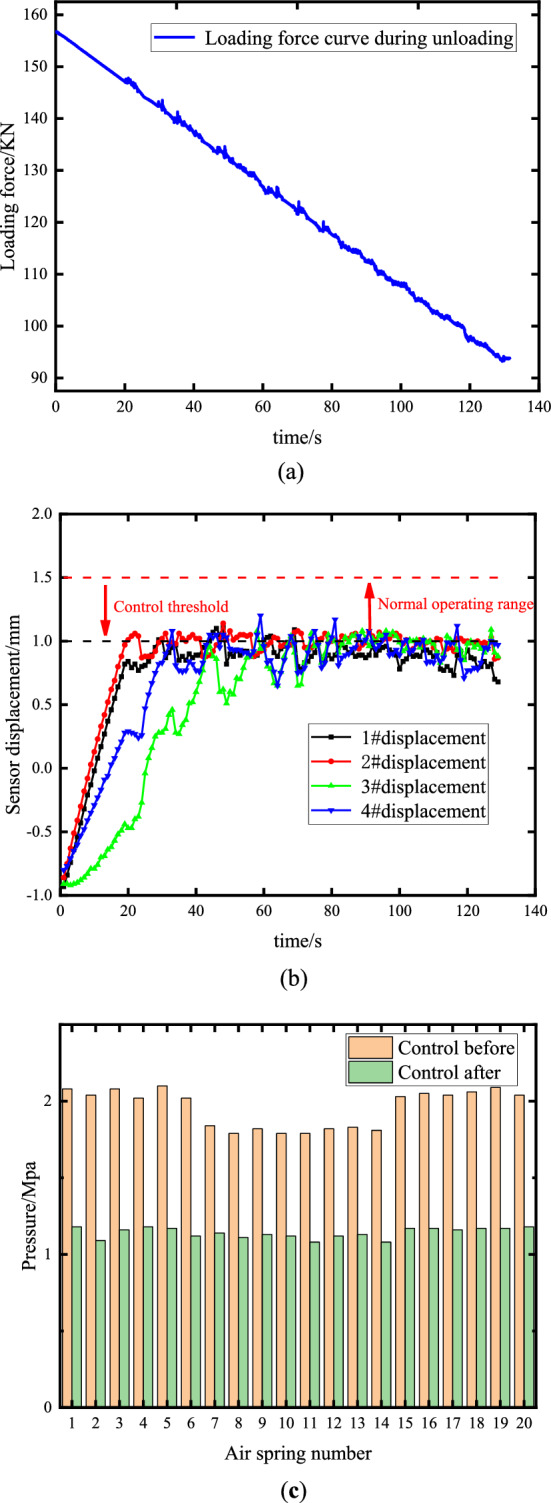
Unloading condition control performance experience. (**a**) Loading force curve during unloading; (**b**) Attitude control curve of raft during unloading process. (**c**) Controlling the pressure distribution of front and rear air spring during unloading.

Firstly, 156 tons of loading force was preloaded to the raft. According to relevant performance indexes, the control accuracy was set to ± 1.5 mm, and the load was slowly unloaded to 94 tons, with a load change of about 40%. The load adaptive and attitude control performance of the proposed algorithm is verified. The uniform variation of the floating raft liquid tank mass is achieved by a linear loading force applied from a hydraulic cylinder. The loading force change curve is shown in Fig. 10a. During unloading, the raft attitude control curve is indicated in Fig. 10b. Before and after control, the air pressure distribution of air spring Q1-Q20 is displayed in Fig. 10c, and the displacement is illustrated in Table 4.

**Table 4:** Unloading condition control performance.

Displacement of measuring point in state before/after control(mm)			
1#	2#	3#	4#
− 0.98/0.69	− 0.93/0.87	− 0.9/0.91	− 0.85/0.96
Load equalization performance before/after control (Maximum deviation of pressure(σ)			
0.17/0.06			

During the unloading process of the four hydraulic cylinders, the center of gravity of the raft frame shifted towards the 1# and 2# measurement points. As a result, there was a delay in the height change of the 3# and 4# measurement points. However, within 40 s, all four measurement points achieved a remarkable control accuracy of 1.5 mm, which demonstrates the control algorithm’s adaptability to variations in the raft's weight and center of gravity. Table 4 indicates that by comparing the maximum pressure deviation before and after control, the air spring vibration isolation system consistently maintains uniform load performance throughout the control process.

## Conclusion

This paper tackles the issue of significant changes in mass on the raft during the operation of the floating raft vibration isolation system. Focus mechanical analysis on system modal frequencies and the attitude change in the raft frame. To address this issue, proposed an adaptive mass change control method and carry out experimental studies. The results demonstrate the effectiveness of this approach as follows:

1. Alterations to the mass on the raft have a considerable impact on the modal frequency of the vibration isolation system, particularly affecting higher-order frequencies. By adaptively adjusting the airbag pressure, low-order frequency fluctuation in the system can be reduced, which enhances the system's low-frequency vibration isolation performance.
2. Changes in mass distribution and the center of gravity cause the floating raft structure to experience significant displacements. By analyzing the raft frame's displacement response, the study finds that pressure distribution adjustment within the airbag can counteract this issue, restoring the raft frame to an equilibrium attitude.
3. This research proposes a control method that centers on raft structure displacement and performs load homogenization optimization during the control process. According to the test results, this control method can automatically adjust to variations of up to 40% of the maximum mass of the raft frame, delivering an attitude control accuracy of 1.5 mm. This enables better load homogenization performance of the vibration isolation system.

## Data Availability

The data used to support the findings of this study are available from the corresponding author upon request.
